# Propolis, A Hope for the Future in Treating Resistant Periodontal Pathogens

**DOI:** 10.7759/cureus.682

**Published:** 2016-07-12

**Authors:** Ambreen Shabbir, Maryam Rashid, Hamid N Tipu

**Affiliations:** 1 Pathology, Prince Sultan Military College of Health Sciences, Dhahran, KSA; 2 Pharmacology, Akhtar Saeed Medical and Dental College, Lahore, Pakistan; 3 Immunology, Armed Forces Institute of Pathology, Pakistan

**Keywords:** propolis, antimicrobial activity, periodontitis, pakistan

## Abstract

Introduction: Periodontitis is one of the most common causes of tooth loss worldwide. Recently, special attention has been paid to natural medication for its treatment. For this purpose, propolis (bee glue) activity has also been investigated. Its antibacterial properties are mainly attributed to flavonones pinocembrin, flavonols galangin and to the caffeic acid phenethyl ester. This study is aimed at evaluating the antimicrobial effects of propolis from Pakistan on 35 clinical isolates of pigmented anaerobic periodontal pathogens.

Methods: This study was conducted in the Microbiology department, University of Health Sciences, Lahore, Pakistan. Pathogens included were *Porphyromonas asaccharolytica* (n=9), *Porphyromonas gingivalis *(n=13), *Prevotella intermedia* (n=9), *Prevotella melaninogenica *(n=4). Minimum inhibitory concentration (MIC) to three antibiotics was obtained by E-test method. All strains were sensitive to amoxicillin plus clavulanic acid and metronidazole, but 100% of *P asaccharolytica* and *P melaninogenica* strains displayed intermediate resistance to tetracycline while 69.2% *P gingivalis* and 100% *P intermedia* strains exhibited complete resistance to tetracycline. Screening for antibacterial activity of propolis extract was done by agar well diffusion assay, and all strains were found sensitive to ethanolic extract of propolis.

Results: MIC was obtained by agar incorporation technique with values ranging from 0.064 to 0.512 mg/ml. It was also noticed that percentage yield of ethanolic extract of propolis prepared from ultrasonic extraction method was higher compared to extract obtained with maceration.

Conclusion: These results indicate that propolis from this region has potent antimicrobial activity against pigmented anaerobic periodontal pathogens. Taking into consideration the increasing resistance in anaerobic bacteria, this effective antimicrobial activity of propolis gives hope in the treatment of oral cavity diseases.

## Introduction

Periodontitis is a biofilm-induced chronic inflammation of periodontium which has been implicated as a risk factor for atherosclerosis, diabetes, and possibly rheumatoid arthritis. Its primary etiology is bacterial biofilm that is critical in initiation and progression of periodontitis, thus inciting a host inflammatory response causing irreversible damage to periodontal tissues and tooth loss if left untreated [1]. Management of periodontitis includes mechanical removal/reduction of this bacterial biofilm by scaling and root planing along with administration of systemic antibiotics and topical antiseptics where required. However, it has been witnessed over the years that indiscriminate use of antibiotics either increases resistance to antimicrobial agents or causes overgrowth of intrinsically resistant pathogens [2]. Recently, alternative medicine has come into the limelight to address this issue, and propolis, amongst other herbs, has been studied extensively.

Propolis (bee glue) is the generic name for an adhesive, complex resinous material collected by honey bees from buds and exudates of various plant sources. After collection, it is enriched with bee saliva and secretions containing enzymes and then used in construction, adaptation, and protection of hives [3]. It is the most powerful chemical weapon of bees against invading bacteria, fungi, viruses, and parasites [4]. Propolis is as old as honey in its use by man since 300 BC. History suggests its use by ancient Egyptians, Persians, and Romans [5]. Roman soldiers carried it as emergency war-wound medicine, Egyptians used it to embalm their dead, Aristotle recommended it to treat abscesses, ancient Greeks called it a “cure for bruises and suppurating sores,” and records from 12th-century Europe describe propolis use for the treatment of mouth and throat infections and dental caries [6-7]. Constituents of propolis vary depending on the area from where it is collected. It has more or less 50% resin and balsam, 30% wax, 10% essential and aromatic oils, 5% pollen, and 5% impurities. The main chemical classes present are flavonoids, phenolics, and aromatic compounds while the volatile oils, terpenes, and bee wax do not contribute significantly to its chemical properties and effects [8]. Its antimicrobial properties are mainly due to flavonones pinocembrin, flavonols galangin, and caffeic acid phenethyl ester; but it is believed that the antibacterial action of propolis depends on synergism of these compounds [9]. Numerous studies have proven its versatile biological activities including anti-inflammatory, immunostimulatory, and antimicrobial activities. It acts against a wide range of bacteria, fungi, yeasts, viruses, and invading larvae [10].

In Pakistan, propolis is being produced along with honey in commercial apiaries. It is scraped off from the walls and frames of beehives, but is considered useless and thus discarded. The reason why propolis in Pakistan is considered worthless is due to a lack of research on its various properties. The aim of this study was to determine whether propolis from this region possessed antibacterial activity against periodontal pathogens, and accordingly educating our beekeepers to value it.

## Materials and methods

This study was approved by the Institutional Review Board and Ethical Committee of University of Health Sciences (UHS), Lahore, Pakistan. Crude propolis samples of two origins were acquired from the National Agricultural Research Council, Islamabad. Sample 1, PS (Propolis Skardu) was collected from Skardu with plant origin *Robinia pseudoacacia *and *Elegnus agustifolia* (Russian olive) while Sample 2, PI (Propolis Islamabad) was collected from the Margalla hills, Islamabad originating from plant source *Acacia modesta. *All samples were from *Apis mellifera* bees. Coarse debris and excessive wax were removed from propolis chunks, and the propolis chunks were ground to powder form. Since most of the active ingredients are soluble in ethanol, we used ethanol (95%) as a solvent. The initial concentration did not exceed 30 % (w/v) due to less efficient and incomplete extraction at higher concentration. Therefore, three parts propolis was added to seven parts of 95% ethanol. Both the propolis powder and ethanol were poured in an Erlenmeyer flask, the top was sealed, and the mixture was shaken briefly.

Two types of extracts were obtained by utilizing two different techniques [11].

1. Ultrasonic extraction (UE): The flask containing the propolis and ethanol mixture was placed in a sonicator at 300W, 25^o^C for 30 minutes.

2. Maceration Extraction: Propolis dipped in ethanol was placed in a dark, cool place for one week and shaken occasionally. Both the solutions were filtered twice using Whatman 125 mm filter paper and were evaporated with a rotary evaporator (Heidolph apparatus).

A propolis paste was obtained and the percentage yield calculated. The ethanolic extracts were standardized by submitting them to a temperature of 50^0^C after which the resin content was diluted with 95% ethanol to obtain a stock solution of 300 mg/ml [12].

### Bacterial strains

A total of 35 clinical isolates were used. Included were *Porphyromonas saccharolytica *(n=9), *Porphyromonas gingivalis *(n=13), *Prevotella intermedia *(n=9), *Prevotella melaninogenica *(n=4). All isolates belonged to a collection of the microbiology laboratory at UHS. For quality control, two reference strains, namely *Porphyromonas gingivalis* (ATCC 33277) and *Bacteroides fragilis* (ATCC 25285), were included in the study obtained from reference center (MicroBioLogics, USA) and identified by morphological, cultural, and biochemical profile (API -20A, bio Merieux, France). These reference strains were included in every cycle of culture and susceptibility testing to monitor the consistency of the procedure.

### Microbiological techniques

**Figure 1:**
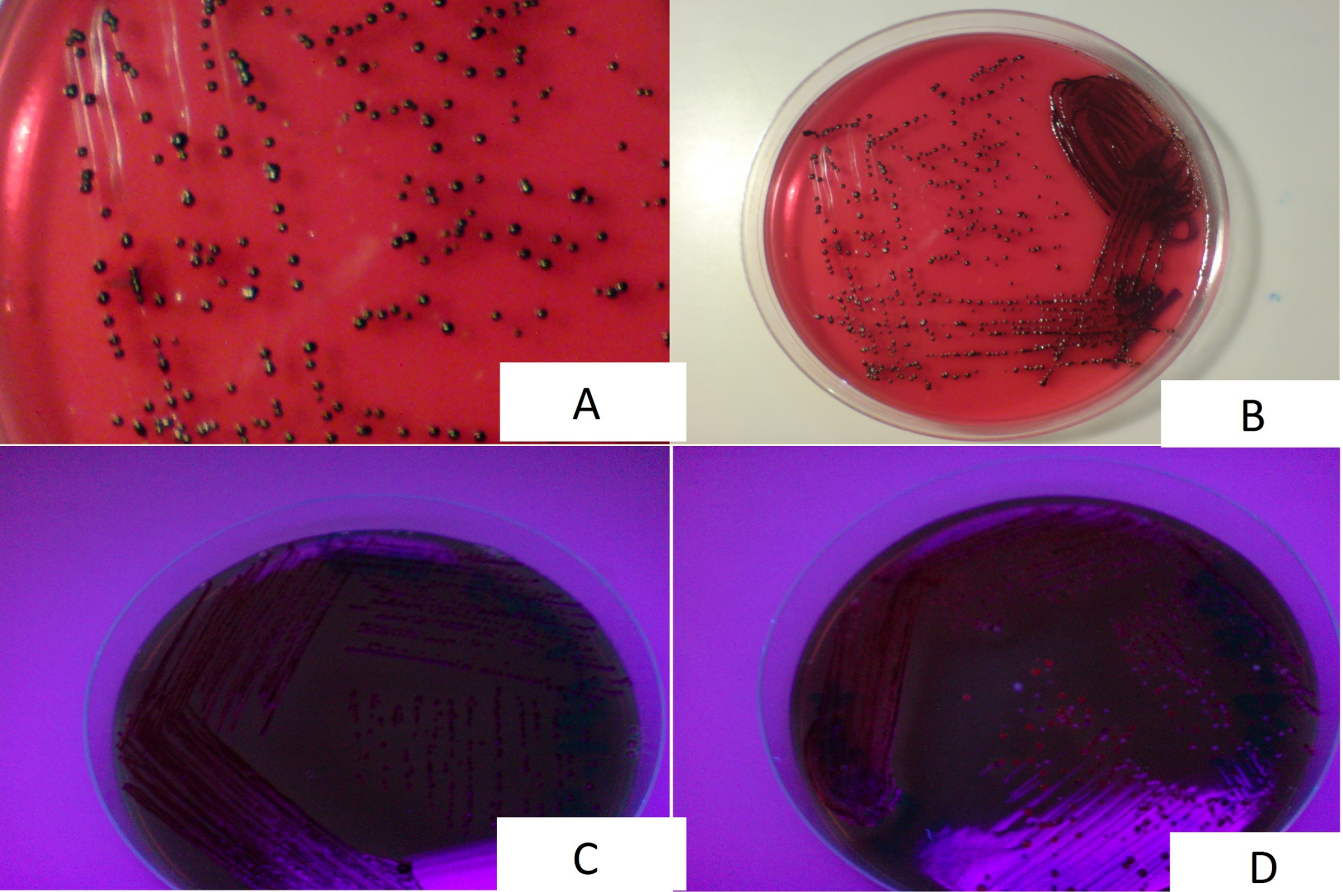
A and B: Dark Pigmented Colonies of Porphyromonas Gingivalis, C: Non-Fluorescent Colonies of Porphyromonas Gingivalis, D: Fluorescent Colonies of Porphyromonas Asaccharolytica

For each experiment, the bacteria were inoculated on anaerobic basal agar (ABA) (Oxoid, UK) supplemented with 5% defibrinated horse blood, incubated at 35^o^C in anaerobic jars for five days. Pigmented colonies were inoculated on the ABA once again to obtain a pure growth and incubated in an anaerobic jar. The same colonies were also plated on blood agar and incubated aerobically at 37°C to confirm their anaerobic nature. The colonies from pure growth were identified up to species level by colonial morphology, gram staining, and API 20A (bio Merieux, France). Identification of *P. gingivalis* was further confirmed and differentiated from other members of the Porphyromonas genus on the basis of florescence and hemagglutination [13]. In contrast to all other species in the genus Porphyromonas, strains of *P. gingivalis* do not give fluorescence under UV light (365 nm) by Woodlight lamp (Crossmedico, Germany) (Figure 1).

E-testing was used to evaluate the susceptibility and minimum inhibitory concentration (MIC). Commercially available antibiotics were used in the form of E-test strips (AB Biodisk, Sweden. Appendix V) in accordance with the Clinical and Laboratory Standards Institute (CLSI) guidelines. The following antibiotics were used: amoxicillin plus clavulanic acid, (amoxicillin plus clavulanic acid) (XL), tetracycline (TC), and metronidazole (MZ).

### Screening of propolis extract for antibacterial properties

Screening of propolis extract for antibacterial properties was done by the agar well diffusion technique. Four samples of propolis extract were each weighed and a stock solution prepared equivalent to 300 mg in 1 ml of ethanol to achieve 30% w/v ethanolic extract of propolis (EEP) [14]. Pure colonies were inoculated with a sterile swab in anaerobic basal broth and concentrations adjusted equivalent to the McFarland scale 0.5. This inocula was plated onto anaerobic blood agar plates in three directions by sterile swabs. Four wells (9 mm diameter) were punched in the plates using a sterile stainless steel borer. Each well was filled with 120μl of EEP (30 % and 15% concentrations) of two different origins obtained with the two techniques. Equal quantities of diluent, i.e., ethanol, were also poured in the wells as negative control. The plates were incubated in an anaerobic atmosphere using Gas Pak anaerobic system envelopes at 35^0^C for 48 hours after which diameters of inhibitory zones around the wells were measured in millimeters with the help of digital calipers (Sylvac, Fowler, Ultra-Call11). The strains were tested in triplicate and mean values of growth inhibition for each strain were taken into account [14].

### Determination of minimum inhibitory concentration

The minimum inhibitory concentration (MIC) was obtained by agar incorporation technique. A series of agar plates each containing a different concentration of EEP were inoculated with up to 35 isolates. After 48 hours incubation at 35^0^C in an anaerobic environment, the MIC was determined by observing the lowest concentration of propolis extract inhibiting visible growth of test isolates. Recommendation for performance of agar dilution method for susceptibility testing of anaerobic bacteria is found in NCCLS M11 standard.

### Modification of the method

This method uses Brucella blood agar with hemin, vitamin K and laked sheep blood. We used anaerobic basal agar that already has these supplements and we added 5% lysed horse blood.

### Controls

Positive growth control plates (without extract) after inoculation were incubated anaerobically, negative growth control plates (without extract) after inoculation were incubated aerobically, purity plates and inoculum control plates were added in the study. Pre and post plates (without extract) were also included to check the viability of our isolates at the start and end of the inoculation procedure. After incubation, positive and negative (contamination) growth control plates were checked, and the reading was recorded.

## Results

The data were analyzed by using SPSS version 16.0. Our first observation was that percentage yield of EEP prepared from UE method was higher as compared to the extract obtained with maceration, suggesting it to be a more efficient extraction method (Table 1).

**Table 1:** Comparison of Percentage Yield of EEP Prepared by Ultrasonic Extraction and Maceration Technique Percentage yield =  weight of dry extract divided by weight of crude propolis x 100

Propolis Sample	Geographical Origin	Plant Source	Technique for Preparation of Extract	Yield (%)
PSM	Skardu	*Robinia pseudoacacia, Elegnus agustifolia*	maceration	31
PIM	Islamabad	*Acacia modesta*	maceration	32
PSU	Skardu	*Robinia pseudoacacia, Elegnus agustifolia*	ultrasonic extraction	37
PIU	Islamabad	*Acacia modesta*	ultrasonic extraction	38

MIC values of three antibiotics, i.e., tetracycline; amoxicillin plus clavulanic acid; and metronidazole, against test isolates were recorded (Table 2).

**Table 2:** MICs of Antibiotics Against Pigmented Anaerobic Periodontal Pathogens by E-Test Breakpoints by CLSI                            Sensitive   Intermediate   Resistant Amoxicillin plus clavulanic acid              ≤4                8               ≥16 Tetracycline                                              ≤4               8               ≥16 Metronidazole                                          ≤8               16              ≥32

Isolates (n)	E-test Strips	Concentration(µg/ml)			
		MIC range	MIC _50_	MIC _90_	
*Porphyromonas asaccharolytica *(n=9)	TC Tetracycline	5.67-7.05	6.3	6.6	
	XL Amoxicillin plus clavulanic acid	0.04-0.10	0.09	0.09	
	MZ Metronidazole	0.04-0.05	0.05	0.05	
*Porphyromonas gingivalis* (n=13)	TC Tetracycline	0.19-19.8	19.3	19.6	
	XL Amoxicillin plus clavulanic acid	0.03-0.16	0.14	0.15	
	MZ Metronidazole	0.05-0.08	0.05	0.06	
*Prevotella intermedia *(n=9)	TC Tetracycline	15-16.33	15	16.24	
	XL Amoxicillin plus clavulanic acid	0.12-0.26	0.13	0.21	
	MZ Metronidazole	0.02-0.04	0.028	0.03	
*Prevotella melaninogenica* (n=4)	TC Tetracycline	7.5-7.73	7.5	7.7	
	XL Amoxicillin plus clavulanic acid	0.19-0.23	0.19	0.2	
	MZ Metronidazole	0.07-0.08	0.08	0.08	

**Figure 2:**
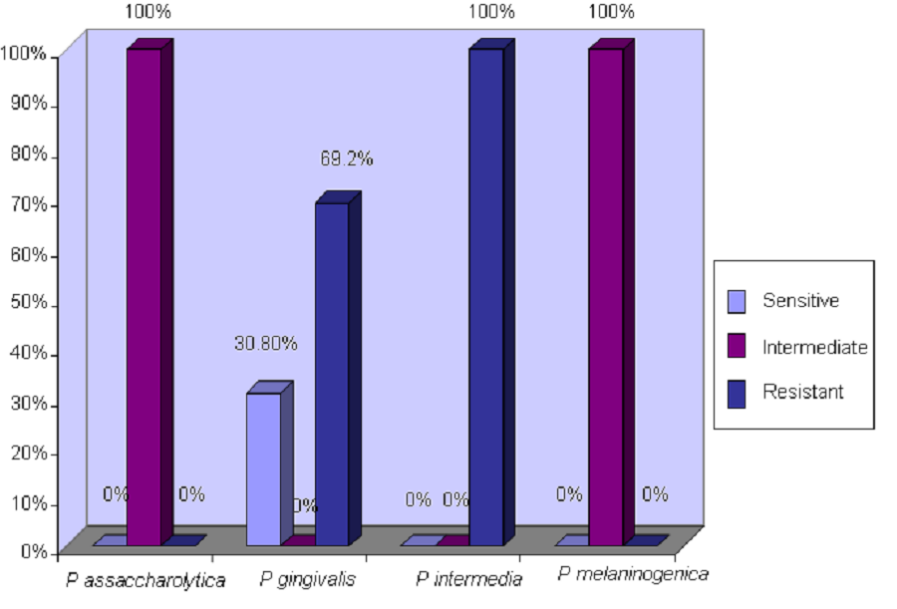
Susceptibility Pattern of Pigmented Anaerobic Periodontal Pathogens to Tetracycline

All isolates were susceptible to amoxicillin plus clavulanic acid and metronidazole. All* P assacchrolytica* and *P melaninogenica *isolates showed intermediate resistance to tetracycline while 69.2% of *P gingivalis* and 100% of *P intermedia* isolates demonstrated complete resistance to tetracycline (Figure 2).

The isolates tested were all susceptible to propolis extract on screening by agar well diffusion assay (Table 3).

**Table 3:** Propolis Extract Against Pigmented Periodontal Pathogens Used in Agar Well Diffusion Technique EEP: Ethanolic extract of propolis PSM: Ethanolic extract of propolis from Skardu prepared by maceration PIM: Ethanolic extract of propolis from Islamabad prepared by maceration PSU: Ethanolic extract of propolis from Skardu prepared by ultrasonic extraction PIU: Ethanolic extract of propolis from Islamabad prepared by ultrasonic extraction Significant difference was observed in zone sizes of different isolates when tested against ethanolic extract of propolis from Islamabad and Skardu prepared by maceration and ultrasonic extraction method. On applying post hoc, p-values for PSM, PIM, PSU, and PIU were <0.005 for AN-33 (EEP conc. 30 %), < 0.01 for AN- 32 (EEP conc.30 %), < 0.01 for AN-32 (EEP conc. 15%), <0.01 for AN-10 (EEP conc. 15%), and <0.01 for AN-17(EEP conc. 15%). Significant p-value ≤ 0.05.

Organism	Propolis Extract in Ethanol, 30% (Zone Size in mm)				Propolis Extract in Ethanol, 15% (Zone Size in mm)			
	PSM	PIM	PSU	PIU	PSM	PIM	PSU	
*Prevotella intermedia* (AN-33)	20.4±1.19	21.8±0.65	19.8±0.37	22.8±0.18	19.2±0.25	20.0±0.34	19.6±0.74	
*Porphyromonas gingivalis* (AN-32)	15.7±0.37	17.2±0.81	14.9±0.65	18.9±0.05	13.7±0.25	14.5±0.49	13.2±0.21	
*Prevotella melaninogenic *(AN-10)	14.8±0.26	17.3±0.37	16.4±0.36	18.3±0.64	14.8±0.26	17.7±0.51	14.9±0.36	
*Porphyromonas asaccharolytica *(AN- 17)	19.5±0.66	20.2±0.38	19.8±0.20	22.8±0.28	17.9±0.36	19.7±0.21	18.5±0.47	
ATCC 33277​ *Porphyromonas gingivalis*	20.7±0.60	18.6±0.28	20.2±0.28	21.8±0.31	17.7±0.30	18.4±0.57	19.2±0.65	
ATCC 25285 *Bacteriodes fragilis*	18.6±0.55	19.8±1.04	18.5±0.5	20.9±0.41	17.5±0.92	18.7±0.52	18.0±0.38	

**Figure 3:**
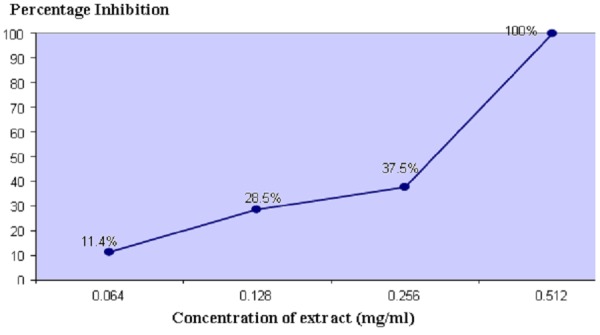
Cumulative Percentage of Anaerobic Periodontal Pathogens Inhibited at Different Concentrations of Propolis Extract

A significant difference was noted in the activity of the four extracts, showing that propolis from different geographical origins has different antibacterial activity on our isolates. The highest activity was observed in EEP from Islamabad prepared by UE method. This extract was further used for determination of MIC by agar incorporation assay. MIC ranged from 0.064 to 0.512 mg/ml (Table 4) (Figure 3). 

**Table 4:** MIC of Ethanolic Extract of Propolis on Isolates Using Agar Dilution Assay

Black Pigmented Pathogens	MIC (mg/ml)		
n=35	MIC Range	MIC _50_	MIC _90_
	0.064-0.512	>0.256	0.51

This verification of antibacterial effect of propolis is not surprising since its primary function is to act as a biochemical weapon by honey bees to protect their hives against foreign pathogens [7].

## Discussion

In the start of our study once the extract was prepared, it was noticed that the percentage yield of EEP prepared from the UE method was higher compared to the extract obtained with maceration. This implied that the UE method is more efficient with regard to percentage yield and is less time consuming as it requires 10 to 30 minutes for maximum extraction while the maceration procedure demands 72 hours to one week for extracting maximum components. A similar study was performed in Bulgaria in 2007 comparing different extraction methods employing three techniques: maceration, UE, and microwave assisted extraction (MAE), and they concluded that compared to maceration extraction, MAE and UE methods provided higher extraction yield, requiring less time and labor [11]. In our study, complete  resistance to tetracycline was demonstrated by the *P gingivalis* and* P intermedia *isolateswhile  intermediate resistance to tetracycline was indicated by both *P asaccharolytica* and *P melaninogenica* isolates. In Brazil, a group of researchers in 2002 concluded that 5 % of their *P intermedia* isolates were resistant to tetracycline (MIC of 32 mg/mL) whereas 30% of the isolates were resistant to clindamycin (MIC ≥ 8 mg/mL) [7]. Another study that came in the limelight in 2003 reported that resistance of pigmented anaerobic pathogens to clindamycin and metronidazole has emerged as *Prevotella intermedia/Prevotella nigrescens *isolates and showed 62.5% susceptibility to benzylpenicillin and 87.5% to both clindamycin and metronidazole [12].

Screening for antibacterial activity of propolis was achieved by agar well diffusion technique using 30% and 15% EEP samples from Islamabad and Skardu prepared by maceration and ultrasonic extraction method (PIM, PSM, PIU, PSU). One organism each out of the different species was tested and was found susceptible to propolis extract as shown in Table 2. ATCC 33277* P gingivalis *andATCC 25285* B fragilis *were also included in screening to monitor quality control and the values correlate with other studies suggesting that our technique was up to the mark. 95% ethanol was used as negative control which gave no zone of inhibition while phenol 6% used as positive control and gave a significant zone. During this procedure, significant difference was observed in zone sizes of different isolates when tested against the ethanolic extracts of propolis from Islamabad and Skardu prepared by different extraction method. On application post hoc, p-values for PSM, PIM, PSU and PIU were <0.005* for AN-33 (EEP conc. 30 %), < 0.01* for AN- 32 (EEP conc.30 %), < 0.01* for AN-32 (EEP conc. 15%), <0.01* for AN-10 (EEP conc. 15%) and <0.01* for AN-17 (EEP conc. 15%); *significant p-value being ≤ 0.05. A research in Bulgaria (2006) on the activity of Bulgarian propolis utilized 30% EEP in which 7 mm wells were filled with 30 or 90 μL propolis extract [11]. The zone of inhibition in our research is greater compared to this study probably because of using more volume of extract (120 μL) in a bigger well (9mm).

For determination of MIC of EEP, we performed agar dilution assay using ultrasonic extract of propolis from Islamabad (PIU) since it demonstrated highest antibacterial activity. The MIC values ranged from 0.064 to 0.512 mg/ml with MIC_50 _being > 0.256 mg/ml and MIC_90 _being 0.512 mg/ml as seen in Table 4 and Figure 2. It is worth mentioning here that our clinical isolates which displayed resistance to tetracycline were susceptible to propolis extract. The reason being that there is not one active ingredient in propolis but many, and its antimicrobial action depends on synergism between its ingredients. That is why no reports of bacterial resistance to propolis have been documented . Although the EEP concentrations that inhibited the bacteria seem rather high compared to the antimicrobial agents used in dentistry, this is expected since they were all crude extracts. These values relate well to a Turkish study published in 2007 in which five propolis samples were collected from four different regions in Turkey and Brazil and were tested against nine ATCC anaerobic isolates. All isolates were susceptible and MIC values ranged from 0.04 to 0.512 mg/ml [9]. In 2005, another research group in Turkey determined the effect of bee propolis on oral pathogens and human gingival fibroblasts. They tested NCTC strains with MIC values from 0.128 mg/ml to 0.256 mg/ml, which is parallel to MIC of our ATCC strains suggesting that our method for MIC assay was up to the mark [10]. One research conducted in Brazil obtained MIC values that ranged from 0.064 to 0.256mg/ml for clinical isolates of* P. intermedia/P. nigrescens *isolates while our MIC range is slightly higher from 0.064 to 0.512 mg/ml. This difference could be due to variation in geographical origin and plant source. They also observed difference between MIC of crude extract and commercial extract on ATCC *P. gingivalis*. The crude extract exhibited less antibacterial activity, with MIC being 0.128 mg/ml, than the commercial one, with MIC of 0.064 mg/ml [7]. In 2010, another study was conducted in Turkey in which the MIC and MBC of propolis samples ranged from 0.4 ­ 0.6 mg/ml to 108.1-186.2 mg/ml, respectively. They stated that *Actinomyces odontolyticus* was the most susceptible strain; whereas *Prevotella intermedia* was least susceptible to all tested propolis samples. We had also tested clinical isolate of *Prevotella intermedia* and found it completely resistant to tetracycline. Meanwhile, it showed the highest zone of inhibition when tested against the propolis sample from Islamabad.

A reason for variability in results is the difference in composition of the propolis being used. A different geographic location with a different plant origin would yield propolis with different properties. Therefore, it is extremely important to mention botanical origin and geographical location of the propolis used in research. Better yet, if the samples are analyzed to determine the variation in ingredients, the differences in the propolis properties can be justified logically.

Regarding the methodology, a number of variables determine the outcome of agar dilution assay. These are pH, temperature, components of medium, size of inoculum and length of incubation. That is the reason why this method demands standardization so that interstudy results can be safely compared. To this end, it is important to develop guidelines for all procedures adopted in evaluating antibacterial activity of propolis.

It is a well-established fact that a single propolis component does not have an activity greater than that of the total extract. Synergism between different compounds seems to be the most important process to explain the antibacterial activity of propolis. Furthermore, there are no reports dealing with bacterial resistance to constituents of propolis, and this information is very significant since the *P. intermedia/P. nigrescens* group may function as an antibiotic resistance gene reservoir and thus influence the success of antibiotic therapy in the oral cavity [7]. The present study regarding the susceptibility of pigmented anaerobic periodontal pathogens to propolis extract clearly demonstrates the antibacterial potential of propolis from Pakistan. Current opinion is that the use of standardized preparations of propolis is safe and less toxic than many other synthetic medicines. Since the anaerobic microflora associated with aggressive periodontitis may be resistant to several antibiotics, this reported antimicrobial activity is of relevance. In the future, propolis may constitute an alternative for treating these pathogens, if safe but strong antibacterial concentrations can be found. 

## Conclusions

The results of our study indicate that propolis from Pakistan has potent antimicrobial activity against pigmented anaerobic periodontal pathogens. Considering increasing resistance in anaerobic bacteria, this effective antimicrobial activity of propolis gives hope in the treatment of oral cavity diseases. The comparison between maceration and ultrasound extraction techniques for extract preparation demonstrates a significant difference between percentage yields of extract. It can be concluded that ultrasonic extraction method is less time consuming and laborious, leading to maximum extraction of components. Propolis ethanolic solutions have been widely used commercially on the market in toothpastes, mouthwashes, etc., but it is still an unofficial drug in pharmacy. A step further should be taken to verify if a dose sufficient to kill the target microorganisms can be reached within the oral cavity, without causing major local or systemic adverse effects. In the future, studies in animal models and clinical trials should be performed for evaluating the use of propolis preparations as a prophylaxis or treatment adjuvant to periodontitis.
